# An Energy Storage Performance Improvement Model for Grid-Connected Wind-Solar Hybrid Energy Storage System

**DOI:** 10.1155/2020/8887227

**Published:** 2020-08-28

**Authors:** Rui Zhu, An-lei Zhao, Guang-chao Wang, Xin Xia, Yaopan Yang

**Affiliations:** College of Energy and Mechanical Engineering, Shanghai University of Electric Power, 2103 Pingliang Road, Yangpu District, Shanghai, China

## Abstract

This study introduces a supercapacitor hybrid energy storage system in a wind-solar hybrid power generation system, which can remarkably increase the energy storage capacity and output power of the system. In the specific solution, this study combines the distributed power generation system and the hybrid energy storage system, while using the static reactive power compensation system and the conductance-fuzzy dual-mode control method to increase output power in stages. At the same time, the optimal configuration model of the wind-solar hybrid power generation system is established using MATLAB/Simulink software. The output power of the microgrid to the wind-photovoltaic hybrid power generation system is calculated by simulation, and the optimization process of each component of the system is simulated. This study mainly uses the static reactive power compensation system and the conductance-fuzzy dual-mode control method to optimize the wind-solar hybrid power generation system. Using MATLAB software simulation verifies the feasibility and rationality of the optimal configuration of the system.

## 1. Introduction

China's 13th Five-Year Plan for Energy Development points out that “the developing speed of renewable energy such as hydropower, wind energy, and solar energy should be accelerated.” Among these energy sources, the development and utilization of wind energy and solar energy has become indispensable in the field of renewable energy. Such forms of renewable energy will gradually replace the position of traditional energy in practical application. To increase the capacity of renewable energy, grid-connected wind-solar hybrid energy storage systems have become popular due to the technological maturity of both forms of power [1].

With the extensive and generalized usage of wind-solar hybrid energy storage system, the impact on the grid by wind-solar hybrid energy storage system cannot be ignored. Owing to unsuitability and unpredictability, the power output of a wind-solar hybrid energy storage system could fluctuate randomly and intermittently [2]. Thus, compared with conventional power sources, such as nuclear and coal-based thermal power plants, renewables can be hard to dispatch on the grid. Considering that the power output of wind-solar hybrid energy storage system should achieve an ideally steady level, dispatchable energy storage is introduced. In this study, we integrate supercapacitor and battery together as an energy storage system. Either the energy capacity or the charge-discharge cycle duration of this technology is superior to either individual super capacitor or individual battery energy storage system [3].

A large number of researches have been done on energy storage system performance optimization of off-grid wind-solar hybrid energy storage system. However, few studies can be found in respect to performance optimization of grid-connected wind-solar hybrid energy storage system with energy storage system.

Wang et al. analytically investigated the exchanger effectiveness and thermal performance of a solar energy storage system [2]. Capasso and Veneri integrated supercapacitors and batteries to achieve high-performance hybrid storage system for electric vehicles [3]. Taking an independent solar power generation system as the research object, Shi proposed an improved fuzzy PID control algorithm to achieve maximum power point tracking [4]. Fuzzy control method and conductance incremental algorithm are combined to enable the working point closer to the maximum power point and reduce the oscillation at the maximum power point [5]. Similarly, conductance increment method and improved fuzzy control method are combined by Zhang et al. [6] to realize the maximum power tracking of photovoltaic power generation system.

This study proposes a detailed model of wind-solar hybrid energy storage system with a supercapacitor and a battery-integrated energy storage system. First, Hybrid Particle Swarm Optimization (HPSO) and Maximum Power Point Tracking (MPPT) fuzzy control system were used to optimize the power output of the hybrid energy storage system. Then, the MATLAB simulation model was set up to validate the enhanced performance of the wind-solar power system. The optimized system is flexible in energy dispatching. The grid-connected wind-solar hybrid energy storage system is able to fully make use of the natural complementarity of wind and solar resources. Moreover, with the conductance-fuzzy dual-mode control algorithm, output energy curve tracking and grid dispatching curve are improved. The cost of energy storage system is reduced greatly. Furthermore, environmental and economic benefits are improved.

## 2. Mathematical Model

This section introduces system structure of the wind-solar hybrid energy storage system. Mathematical models are also given with respect to photovoltaic cell, wind turbines, supercapacitors, output power of inverters, and converters.

### 2.1. System Structure

**Figure 1:**
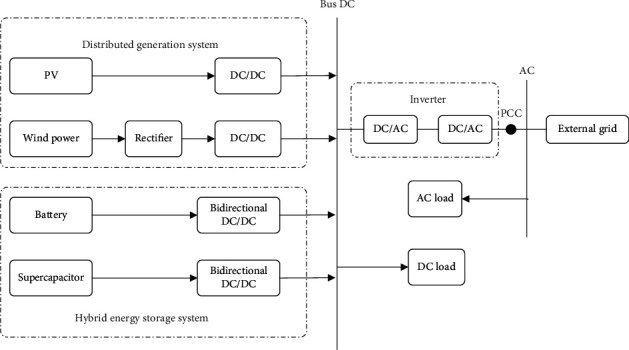
Diagram of the grid-connected wind-solar hybrid energy storage generation system.

Figure 1 demonstrates the structure of the wind-solar hybrid energy storage system. The system consists of wind turbines, inverters, DC bus and AC bus, PV array, and a hybrid energy storage system [7]. The distributed power generation system generates electrical energy, which reaches the inverter through the DC bus and then is converted into alternating current by the inverter [8]. Hybrid energy storage systems regulate energy to ensure that the power generation system provides highly stable electrical power to the load and grid [9].

### 2.2. Mathematical Model for Photovoltaic Cell

PV cell is the most basic unit in a solar power system. Its working principle is similar to P–N junction [10]. The output power relationship between photovoltaic cells is

(1) $$\begin{array}{c} P=\text{IV}=\left(I_{\text{ph}}-I_{d}-I_{\text{sh}}\right)V=\left\{I_{\text{ph}}-I_{0}\left[e^{\left(\left(V+\text{IR}s\right)|/|A\right)}-1\right]-\frac{\left(V+IR_{s}\right)}{R_{\text{sh}}}\right\}V=\left[I_{\text{ph}}-I_{0}e^{\left(\left(V+\text{IR}s\right)|/|A\right)}-I_{0}-\frac{\left(V+{\text{IR}}_{s}\right)}{R_{\text{sh}}}\right]. \end{array}$$

In this formula, *I* is the output current. *I*_*d*_ is the current of the diode in this equivalent circuit. *I*_ph_ is the current generated by the PV array. *I*_sh_ is the current flow by the parallel resistance. *A* is the ideal factor for P–N junction. *V* is the terminal voltage of the loads. *R*_*s*_ is the series resistance. This formula can be simplified as the following formula because R_sh_ is infinitely large and *R*_*s*_ is infinitely small:

(2) $$\begin{array}{c} P=\text{IV}=\left[I_{\text{ph}}-I_{0}e^{\left(\left(V+\text{IR}s\right)|/|A\right)}-I_{0}\right]V. \end{array}$$

**Figure 2:**
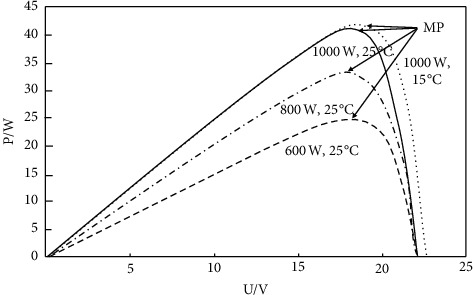
Photovoltaic cell output power characteristic curve.

With the influence of external conditions, a solitary maximum output power point will appear (MP) [11].  Figure 2 shows a typical PV P–U characteristic curve.

### 2.3. Mathematical Model for Wind Turbines

Data analysis shows that the Weibull distribution can be applied to the dataset in this article [8]. Thus, the Weibull distribution is adopted to evaluate the probability density of the wind speed data. The formula is shown as follows [12]:

(3) $$\begin{array}{c} \phi_{w}=\left(\frac{k}{v}\right){\left(\frac{v}{c}\right)}^{k-1}e^{-\left[{\left(v/c\right)}^{k}\right]}. \end{array}$$

The probability density distribution function is obtained by integrating function in (3) in the effective interval:

(4) $$\begin{array}{c} \phi_{w}=1-e^{-\left[{\left(v/c\right)}^{k}\right]},\;\left(k>0,c>1\right). \end{array}$$

In the previous formulae, *k* determines the shape of the curve. *c* is scaling parameter, and *c* is usually considered average wind speed.

To define the value for output power *P*_rwind_, linear interpolation calculation is introduced by the HOMER simulation software [13, 14], as shown in the following equation:

(5) $$\begin{array}{c} P_{\text{rwind}}=\left\{\begin{array}{c} P_{0}+V\frac{P_{1}-P_{0}}{V_{1}}\left(0<V<V_{1}\right),\\ P_{1}+V\frac{P_{2}-P_{1}}{V_{2}-V_{1}}\left(V_{1}<V<V_{2}\right),\\ \vdots \\ P_{n}+V\frac{P_{n}-P_{n-1}}{V_{n}-V_{n-1}}\left(V_{n-1}<V<V_{n}\right). \end{array}\right. \end{array}$$

**Figure 3:**
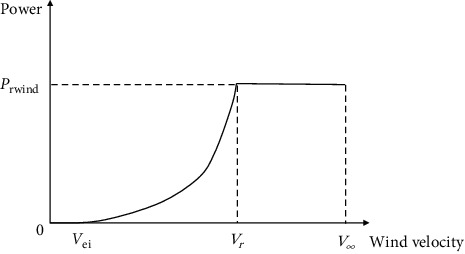
Wind turbine output power characteristic curve.

Figure 3 shows the power curve [15].

### 2.4. Mathematical Model for Supercapacitors

In the course of this work, capacitive values and voltages correspond to both sides of the capacitor, and Δ*W* represents the supercapacitor energy change in the equivalent circuit (Δ*W* is the integration of *P*_*c*_ (*t*) with *t*) within the interval [*t* (*n *−* *1), *t* (*n* + 1)]. The energy change of the capacitor is as follows [16]:

(6) $$\begin{array}{c} \Delta W=\frac{1}{2}u_{c}^{2}\left(t_{n+1}\right)\cdot C\left(t_{n+1}\right)-\frac{1}{2}u_{c}^{2}\left(t_{n-1}\right)\cdot C\left(t_{n-1}\right)=\int_{t_{n-1}}^{t_{n+1}}P_{C}\left(t\right)\text{d}t. \end{array}$$

**Figure 4:**
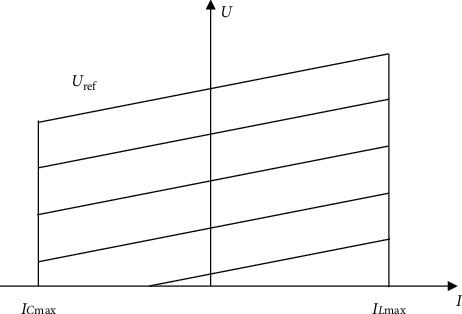
Voltage-current characteristics of SVG.

*u*_*c*_ (*t*) is capacitor voltage. The series resistance of the capacitor is *R*_*s*_. The measured value for the capacitor is represented by *C* (*t*). The series resistance voltage is *u*_*s*_ (*t*). According to Figure 4, the instantaneous power of the capacitor is expressed by the total instantaneous power and the instantaneous power of the resistance:

(7) $$\begin{array}{c} P_{C}\left(t\right)=u\left(t\right)\cdot i\left(t\right)-i^{2}\left(t\right)\cdot R_{s}. \end{array}$$

*R*_*s*_ is the series resistance, *i* (*t*) is the current flow through the capacitor, and *u* (*t*) is the voltage of the capacitor. The integration is calculated by using Simpson's Rule.

(8) $$\begin{array}{c} \Delta W=\frac{1}{2}{\left[u_{c}\left(t_{n+1}\right)-R_{s}i\left(t_{n+1}\right)\right]}^{2}\cdot C\left(t_{n+1}\right)-\frac{1}{2}{\left[u_{c}\left(t_{n-1}\right)-R_{s}i\left(t_{n-1}\right)\right]}^{2}\cdot C\left(t_{n-1}\right). \end{array}$$

*R*_*s*_ is constant, and *u* and *i* can be measured.

### 2.5. Mathematical Model for Output Power of Inverters

The power on the load mainly comes from the battery inverted power. The output power of inverter is *P*_out_ and the input power is *P*_in_. Thus, the efficiency *η* is calculated as [15]

(9) $$\begin{array}{c} \eta =\frac{P_{\text{out}}}{P_{\text{in}}}\text{.} \end{array}$$

The input power equals the output power and the power lost:

(10) $$\begin{array}{c} P_{\text{in}}=P_{\text{out}}+P_{\text{ioss}}=p_{0}+kp^{2}. \end{array}$$

We can obtain

(11) $$\begin{array}{c} \eta =\frac{p}{p+p_{0}+kp^{2}}=1-\frac{p_{0}+kp^{2}}{p+p_{0}+kp^{2}}. \end{array}$$

In the previous formula, *p* = *P*_out_/*P*_in_ (*p* is inverter and *P*_in_ is the rated power of inverter); the expression equation for *P*_0_ with *k* is

(12) $$\begin{array}{c} p_{0}=\frac{9}{11}{\left(\frac{10}{9\eta_{10}}-\frac{1}{9\eta_{100}}-1\right)}^{2}. \end{array}$$

The efficiency of the inverter at 10% voltage is *η*_10_, and the efficiency of the inverter at 100% voltage is *η*_100_. These specifications are given by the following equation

(13) $$\begin{array}{c} k=\frac{1}{\eta_{100}}-p_{0}-1\text{.} \end{array}$$

### 2.6. Mathematical Model for Converters

Converters are able to convert power from AC bus to DC bus. The bidirectional converter is expressed as follows [17]:

(14) $$\begin{array}{c} P_{\text{con},\text{AC}}=\left\{\begin{array}{cc} R_{\text{inv}}\cdot \eta_{\text{inv}}, & P_{\text{con},\text{DC}}>R_{\text{inv}},\\ P_{\text{con},\text{DC}}\cdot \eta_{\text{inv}}, & 0<P_{\text{con},\text{DC}}<R_{\text{inv}},\\ \frac{P_{\text{con},\text{DC}}}{\eta_{\text{rec}}}, & -R_{\text{rec}}<P_{\text{con},\text{DC}}\leq 0,\\ -\frac{R_{\text{rec}}}{\eta_{\text{rec}}}, & P_{\text{con},\text{DC}}<-R_{\text{rec}}. \end{array}\right. \end{array}$$

*P*_con, AC_ is the power on the AC side. Positive value indicates inverting, whereas a negative number indicates rectifying. *P*_con, DC_ refers to the total power on the DC side; *R*_rec_ refers to the maximum power while the inverter is at the rectifying period, which is the rated capacity.

## 3. Grid-Connected Wind-Solar Hybrid Energy Storage System

In this section, the static reactive compensation system is first provided. Afterwards, an improved MPPT optimization is presented based on conductance-fuzzy dual-mode control method.

### 3.1. Static Reactive Compensation System

The stationary reactive compensator can make the grid stability control accurate, have better control, and enable rapid response. According to reactive power theory, Static Var Generator (SVG) unit and the grid are free of loss in power exchange; then the total instantaneous power of the grid is fixed and does not produce energy loss in the compensator [18].

#### 3.1.1. Static Reactive Compensator SVG Mathematical Model

The capacity of the SVG is determined by the voltage change of the bus. Power flow calculation method is adopted. To ensure that the power system is 1 ≥ cos*ϕ* ≥ 97.0%, parallel capacitors must be added as compensation; then the power factor of the system is shown in the following [19]:

(15) $$\begin{array}{c} \cos\phi =\frac{P}{S}=\frac{P_{1}}{\sqrt{P_{1}^{2}+{\left(Q_{C}+Q_{C1}+Q_{C2}-Q_{1}\right)}^{2}}}. \end{array}$$

The capacity of the wind farm is *S*; *Q*_*C*2_ refers to reactive power source. *Q*_*C*1_ refers to reactive power source; *Q*_*C*1_ stands for capacitive charging power of the line. P_1_ and *Q*_1_, respectively, indicate the wind farm's active or reactive power before compensation. The compensation capacity is shown in the following [20, 21]:

(16) $$\begin{array}{c} Q_{C}=P_{1}\left(\tan\phi_{1}-\tan\phi_{2}\right)=P_{1}\left(\sqrt{\frac{1}{\cos\phi_{1}}-1}-\sqrt{\frac{1}{\cos\phi_{2}}-1}\right), \end{array}$$

where cos*ϕ*_1_ is the system power factor before compensation and cos*ϕ*_2_ is the system power factor after compensation. According to Figure 4, as the grid voltage reference value changes, the SVG voltage-current characteristic curve appears to fluctuate [18]. The output inductive current IL or the output capacitive current IC is controlled by adjusting the AC side voltage value.

Figure 4 shows that the SVG operating range is similar to a rectangle with the same width. The Static Var Compensator (SVC) runs in the inverted triangle whose width is continuously reduced from top to bottom.

#### 3.1.2. Control Strategy for SVG

According to the SVG mathematical model, the MPPT is designed as a double closed-loop controller, and the external loop voltage controller adopts a PI (proportional integral) regulator. The input values are the DC side voltage command Uc^*∗*^ and the Point of Common Coupling (PCC) point voltage command U_PCC_^*∗*^, which generate reference signals for the current inner loop. The SVG dual closed-loop control system diagram of iq^*∗*^ and id^*∗*^ is as follows.

**Figure 5:**
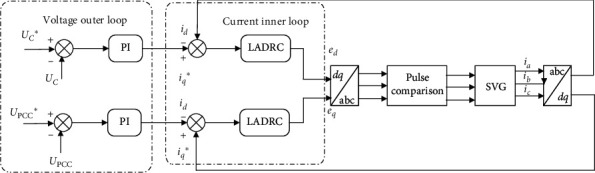
SVG double closed-loop control system diagram.

In Figure 5, the DC side voltage command Uc^*∗*^ and the PCC point voltage command U_PCC_^*∗*^ of the outer ring, respectively, generate the reference signals iq^*∗*^ and id^*∗*^ of the current inner loop through the PI controller. The two reference signals correspond to the two voltages of the outer ring and the Linear Active Disturbance Rejection Control (LADRC) of the inner ring. The output of the device is a voltage pulse signal.

To ensure the stability of the voltage value, the system needs to add voltage closed-loop control to achieve the purpose of controlling the bus voltage to a given value and the system power factor ≥0.95.

#### 3.1.3. SVG Effect on Output Power under Random Wind

Large voltage fluctuations and flicker of wind power may be caused by random wind disturbances, which have serious impact on the quality of wind power. When the system capacity is small, the power quality may be a factor limiting the installed capacity. Therefore, the SVG affects random wind. The study of voltage fluctuations is important and has great significance [22].

**Figure 6:**
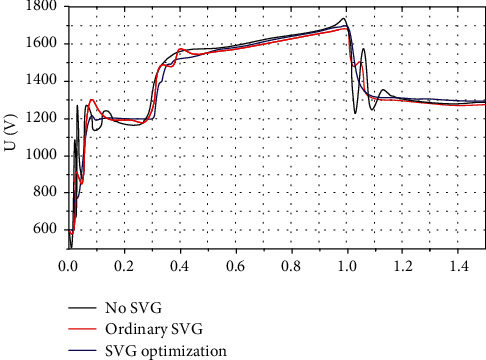
VPCC comparison with or without SVG controller.

**Figure 7:**
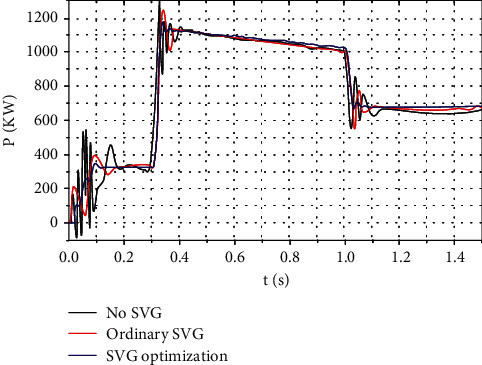
SVC's effect on output power under random wind.

Simulation parameters: wind speed *V* = 10 m/s and random wind ±2 m/s, as shown in Figures 6 and 7. The results show that, in wind farms, SVG devices can remarkably reduce the output power fluctuation caused by random wind.

### 3.2. Improved MPPT Optimization of the Conductance-Fuzzy Dual-Mode Control Method

In this subsection, we first introduce the basic principle of conductance increment method using particle swarm optimization algorithm. Secondly, the fuzzy control algorithm is briefly presented. Finally, the improved conductance-fuzzy dual-mode control method is developed.

#### 3.2.1. Basic Principle of the Conductance Increment Method Based on Particle Swarm Optimization Algorithm

The principle of traditional conductance increment method is a characteristic of the p otovoltaic cell itself, which does not fluctuate with external changes in the environment, and the fluctuation is small after reaching the steady state. Photovoltaic characteristic curve is used as the basis for determining whether the operating condition of the photovoltaic panel is at the maximum power point [23].

**Figure 8:**
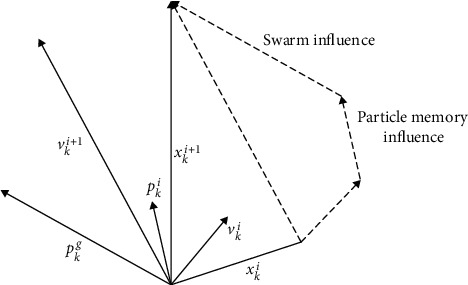
Velocity and position update in PSO.

The working principle of particle swarm optimization algorithm is as follows: Consider *K*-dimensional search space, the number of particles is *n*, and the position and velocity of the No.i particle are *x*_*i*_=(*x*_1_^*i*^, *x*_2_^*i*^,…, *x*_*k*_^*i*^), *V*_*i*_=(*V*_1_^*i*^, *V*_2_^*i*^,…, *V*_*k*_^*i*^). The velocity and position of the particles are then updated according to (17) and (18) [24, 25] as Figure 8:

(17) $$\begin{array}{c} v_{k}^{i+1}=\rho v_{k}^{i}+\vartheta_{1}\text{rend}\frac{p_{k}^{i}-x_{k}^{i}}{\Delta t}+\vartheta_{2}\text{rend}\frac{p_{k}^{g}-x_{k}^{i}}{\Delta t}, \end{array}$$

(18) $$\begin{array}{c} x_{k}^{i+1}=x_{k}^{i}+v_{k}^{i+1}, \end{array}$$

where *p*_ik_ is the optimal position of the *i*-th particle at time *k*, *p*_gk_ is the optimal position of the particle group *k*, *ρ* is the inertia factor, *θ*1 is the local confidence parameter, and *θ*2 is the self-confidence parameter of the particle group.

The workflow of the HPSO algorithm involves using the predicted output power of the neural network as the particle swarm, and then obtaining the inverter switching at the maximum power point by the PSO algorithm [26, 27].

The improved conductance increment method based on the particle swarm optimization algorithm changes the step size of the conductance increment method according to the relationship between the particle spacing and its minimum threshold and achieve the tracking of the maximum output power of the system [28].

#### 3.2.2. Fuzzy Control Algorithm

**Figure 9:**
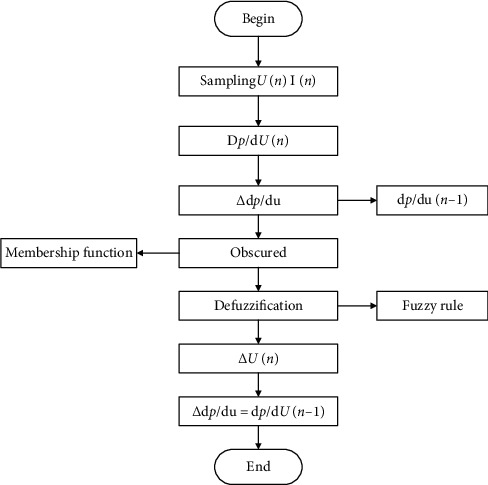
Diagram of the fuzzy control method MPPT control process.

Fuzzy control is a controller designed according to people's operating experience. It does not need to establish a specific mathematical model. The specific control flow principle is determining the controller's membership function and control rules by inputting the number and characteristics of the individual and then selecting the appropriate one. The control method is cleared and the entire control is completed, as shown in Figure 9.

Given that the fuzzy control method does not require a specific mathematical model, the fault tolerance can be very strong. However, the fuzzy processing of information leads to a decrease in system control precision and in dynamic quality.

#### 3.2.3. Improved Conductance-Fuzzy Dual Mode Control Method

**Figure 10:**
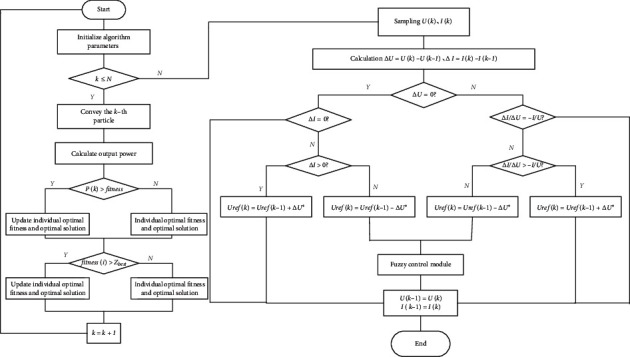
Improved conductance-fuzzy dual-mode control algorithm flow chart.

Given that a single particle swarm optimization conductance increment method or fuzzy algorithm cannot guarantee the accuracy of system control and the time to reach a steady state, an improved mixed control algorithm MPPT is proposed, as shown in Figure 10.

The particle swarm optimization algorithm is used to globally optimize the output power characteristic curve of the system, and the fuzzy controller is used to change the step size in the conductance increment algorithm to locally optimize the system output power curve. When the *E* value is small when the operating point is close to the maximum power point, the fuzzy controller outputs a smaller step size to improve the accuracy at steady state. When the distance from the maximum power point is large, the *E* value is larger, and the fuzzy controller outputs a larger step size to achieve the tracking of the maximum power point [29].

#### 3.2.4. Simulation Example of Environmental Parameters Linear Variation

**Figure 11:**
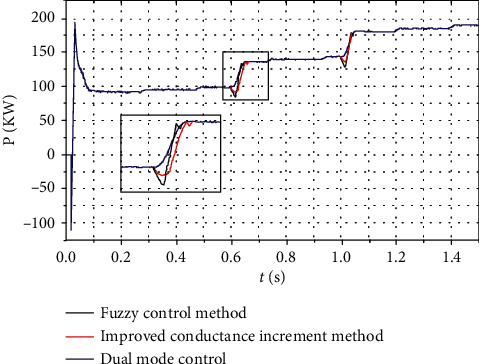
Power changes in the case of environmental changes.

Assume that the environmental parameters are stable and will not change suddenly. Therefore, the test condition in this study is the increase of light intensity from 600 W/m^2^ to 1000 W/m^2^. The particle swarm optimization conductance increment method, the conductance-fuzzy hybrid control method, and the fuzzy control method are simulated.  Figure 11 shows the result [30].

As can be seen from Figure 11, the three algorithms can achieve the tracking of the maximum power point under any circumstances. Whether the fixed condition or loose condition has no effect on the result, compared with the traditional fuzzy control method, the conductance-fuzzy dual-mode control method *T* and Δ*T* designed in this study have different degrees of reduction, and the time for finding the maximum power point is greatly saved. The improved conductance-fuzzy dual-mode control algorithm achieves faster steady-state time and higher tracking accuracy than the particle swarm optimization algorithm.

## 4. Simulation Verification of Stability of the Grid-Connected Wind-Solar Hybrid Energy Storage System

To test the feasibility and effectiveness of the proposed control strategy, a simulation of the load-increasing state of the grid-connected wind-solar complementary system is established.

**Figure 12:**
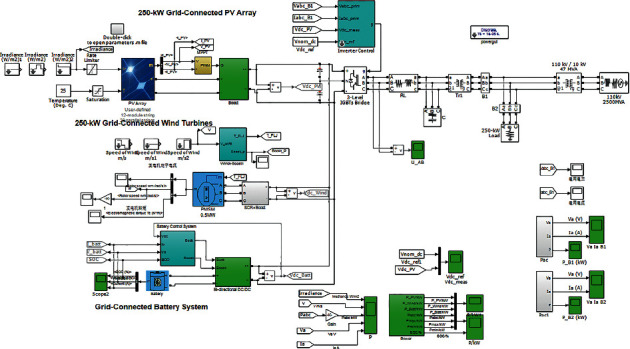
Overall simulation structure of the grid-connected wind and solar hybrid system.

In this study, MATLAB/Simulink is used to build the model for system simulation as shown Figure 12. The solar panel and boost chopper circuit parameters in the simulation are set as follows: the solar panel illumination intensity is reduced from 1000 W/m^2^ to 600 W/m^2^ and then increased to 800 W/m^2^, and the wind speed is increased from 8 m/s to 10 m/s and then drops to 8 m/s. Circuit input and output capacitances are 500 *μ*F, inductance is 5 mH, and load is 25 Ω.

**Figure 13:**
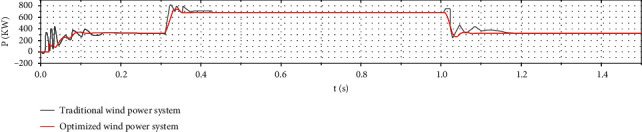
Comparison of output power between traditional and optimized wind turbines.

**Figure 14:**
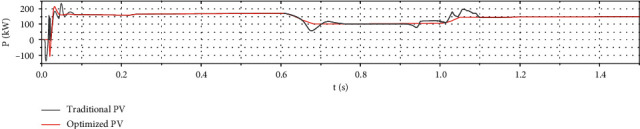
Comparison of output power between traditional and optimized photovoltaic generators.

Figures 13 and 14 show that the optimized photovoltaic power generation system and the wind power generation system reach the steady state faster than the conventional power generation system. The fluctuation is smaller, indicating that the optimized system tracking is more accurate and faster.

**Figure 15:**
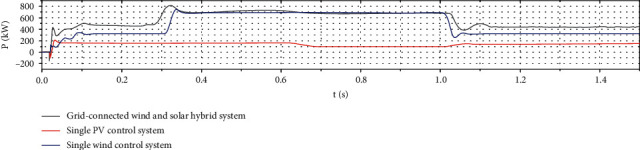
Comparison of output power from single power generation systems with grid-connected wind power systems.

As Figure 15 shows, when the initial condition light intensity is 1000 W/m^2^ and the wind speed is 8 m/s, the optimized wind-solar complementary system tracks the extreme value of the output power and reaches the steady state for approximately 0.16 s. Controlling the photovoltaic system takes approximately 0.05 s to reach the steady state. The traditional wind power system needs approximately 0.115 s to reach the steady state, but the tracking error of the former is approximately 0.23%, and the tracking error of the latter two is 1-2 times of the former. At this stage, the output power of the wind-solar complementary system has a longer steady-state time but less curve fluctuation. At 0.3 s, the ambient wind speed is increased from 8 m/s to 10 m/s. At the same time, the fluctuations of the output power curves of the two systems have different degrees of fluctuation. The optimized wind-solar complementary system needs approximately 0.073 s to reach the steady state again. The tracking speed is faster and the curve fluctuation is smaller. The traditionally controlled wind power generation system needs 0.09 s to reach the steady state, and the curve fluctuates greatly. At 0.6 s, ambient light intensity is reduced from 1000 W/m^2^ to 600 W/m^2^. After optimization, the required time for the wind-solar complementary system to reach steady state is approximately 0.05 s. The duration is approximately 0.089 s, and its tracking error is 400% higher than that of the optimized wind-solar complementary system. The fluctuations of the output power curves of the two are different. At 1 s, ambient light intensity is increased from 600 W/m^2^ to 800 W/m^2^, the wind speed is reduced from 10 m/s to 8 m/s, and the time required for the wind-solar complementary system to reach steady state is optimized with conventional control photovoltaic systems and conventional control. The length of time required for wind power to reach steady state is comparable, but the output curve of the former remarkably fluctuates less than the latter two. According to the whole tracking process, as the optimization degree progresses, the output power curve of the wind-solar complementary system gradually becomes gentle, which is half of the traditional control wind system and one-third of the traditional control photovoltaic system. The average tracking time of the complementary system can be increased by approximately 90% compared with the other two single-power generation systems. Therefore, the optimized performance of the wind-solar complementary system is better.

## 5. Conclusion

This paper focuses on the wind-solar hybrid energy storage generation model. A step-by-step method is presented to optimize the capacity of the wind-solar storage system with the supercapacitor energy storage device. The static wind compensation system and the conductance-fuzzy dual-mode control method are applied to optimize energy storage capacity and power stability. The optimal configuration model of the hybrid energy storage system is calculated and analyzed based on MATLAB. The main conclusions can be drawn as follows:

1. The introduction of a static wind compensation device can stabilize the voltage of wind farms and reduce the influence of power fluctuation on network side voltage. Using MATLAB/Simulink simulation, it is proved that the output power curve of the wind power generation system is smaller than that of the conventional wind power generation system. When the wind speed changes, the power reaches a stable and faster oscillation.
2. The particle group optimization conductivity increment algorithm, conductivity-fuzzy dual-mode control, and traditional fuzzy control are simulated and verified under the conditions of different light intensity. The results show that the conductivity-fuzzy dual-mode control algorithm can reach steady state faster with higher accuracy. In addition, the algorithm improves battery life and reduces power consumption.
3. By comparison, it is found that when the power supply mode of the photovoltaic power generation system or the wind power generation system is solely adopted, the load fluctuation will cause insufficient power supply. The combination of hybrid energy storage system and wind-solar complementary power system verifies the microgrid power supply mode. This power supply mode complements different energy sources, which is one of the effective ways to ensure compliance with requirements.

In the future, it is a promising direction to combine intelligent algorithms with the MPPT, in order to achieve high accuracy and high-intelligence level. On the other hand, energy management of hybrid energy storage systems should also be further optimized, such as the charge and discharge control between batteries and supercapacitors.

## Data Availability

The data used to support the findings of this study are available from the corresponding author upon request.
